# Oxometallate-Based Ionic Liquid Catalyzed CO_2_-Promoted Hydration of Propargylic Alcohols for α-Hydroxy Ketones Synthesis

**DOI:** 10.3390/ijms26010062

**Published:** 2024-12-25

**Authors:** Yuankun Wang, Chongli Wang, Weidong Lin, Qin Wang, Baisong Li, Cheng Chen, Ye Yuan, Francis Verpoort

**Affiliations:** 1 State Key Laboratory of Advanced Technology for Materials Synthesis and Processing, Wuhan University of Technology, Wuhan 430070, China; 331412@whut.edu.cn (Y.W.); 359414@whut.edu.cn (C.W.); 359286@whut.edu.cn (W.L.); 359705@whut.edu.cn (Q.W.); 359251@whut.edu.cn (B.L.); chengchen@whut.edu.cn (C.C.); francis.verpoort@ghent.ac.kr (F.V.); 2 School of Materials Science and Engineering, Wuhan University of Technology, Wuhan 430070, China

**Keywords:** CO_2_, ionic liquid, catalysis, α-hydroxy ketones

## Abstract

α-Hydroxy ketones are a crucial class of organic compounds prevalent in natural products and pharmaceutical molecules. The CO_2_-promoted hydration of propargylic alcohols is an efficient method for the synthesis of α-hydroxy ketones. Herein, an ionic liquid (IL) was designed to catalyze this reaction individually under atmospheric CO_2_ pressure, volatile organic solvents, and additives. This IL, constructed from the molybdate anion, can be recycled from industrial (NH_4_)_2_MoO_4_ production wastewater, demonstrating its high tolerance to catalytic environments and significant potential for practical applications. To our knowledge, this is the first instance of an oxometallate-based IL catalyst being utilized for the CO_2_-promoted hydration of propargylic alcohols. Further mechanistic studies revealed the bifunctionality of this IL in activating both CO_2_ and substrates.

## 1. Introduction

Carbon dioxide (CO_2_), as one of the primary greenhouse gasses, is a key factor contributing to climate change [1,2]. In recent years, global CO_2_ emission has been continuously increasing, leading to a gradual rise in worldwide temperature [3,4]. On the other hand, the advantages of CO_2_, such as non-toxicity, renewability, abundance, and environmental friendliness [5,6], have led to its widespread application in the synthesis of organic compounds [7,8]. Among them, catalytic conversion of CO_2_ for the synthesis of α-hydroxy ketones plays a significant role in this field. α-Hydroxy ketones are crucial organic compounds found in natural products and are key structural components of valuable chemicals [9,10] like taxifolin [11], and hypothemycin [12]. However, compared to traditional C1 sources like carbon monoxide and phosgene, CO_2_ molecules are thermodynamically stable and kinetically inert due to the fact that carbon atoms are in their highest oxidation state [13]. Therefore, the transformation of CO_2_ generally requires high-energy starting materials, low-energy target products, as well as harsh reaction conditions (high temperature, elevated CO_2_ pressure, etc.). Consequently, the activation of CO_2_ under mild conditions is very important for its transformation. Catalysts used for CO_2_ activation mainly include transition metal salts [14,15,16,17], N-heterocyclic carbenes [18,19], ionic liquids (ILs) [20,21], and organic bases [22,23]. Particularly, ILs are a series of molten salts that are liquid at or near room temperature [24]. These compounds can be easily tailored to modulate their physical and chemical characteristics by tuning the structures of ions [25,26]. Common cations employed to construct ILs include quaternary ammonium cations, quaternary phosphonium cations, alkylpyridines, and alkylimidazoles, while corresponding anions can be inorganic, organic, or oxometallate anions [26]. ILs have distinct physicochemical properties, such as low melting points, low vapor pressures, wide electrochemical windows, and customizable catalytic activity [27,28,29]. Consequently, they exhibit broad potential applications in CO_2_ utilization, such as the activation of CO_2_ molecules, CO_2_ capture and adsorption, and the conversion of CO_2_ into fuels and fine chemicals [26,30,31].

In recent years, the increasing attention to oxometallate-based ionic liquids (OM-ILs) has driven their widespread application in catalytic conversion processes [32,33]. Benefiting from their structural diversity and stability, oxometallates exhibit exceptional redox properties, showing vast potential in catalytic chemistry, medicinal chemistry, electrochemistry, and material science [34,35]. OM-ILs typically consist of anions containing high-valent transition metal compounds, such as tungsten, niobium, molybdenum, and vanadium, paired with quaternary ammonium, quaternary phosphonium, or imidazole cations [36,37]. The oxygen-rich structure of oxometallate anions renders the oxygen atoms negatively charged, enhancing their nucleophilicity and enabling the activation of CO_2_ molecules through the formation of adducts [38,39].

In recent years, significant progress has been made using OM-ILs as catalysts in CO_2_ conversion [33]. He et al. utilized [(*n*-C_7_H_15_)_4_N]_6_[α-SiW_11_O_39_Co] (0.1 mol%) as an IL catalyst for the synthesis of propylene carbonate from cyclopropane and CO_2_ [40]. They achieved a yield of 97% of propylene carbonate at 3.5 MPa and 150 °C under solvent-free conditions. Chen and colleagues utilized [(*n*-C_7_H_15_)_4_N]_6_[GeW_11_MnO_39_] (0.1 mol%) for the catalytic synthesis of propylene carbonate from propylene oxide and CO_2_ under conditions of 3 MPa and 150 °C [41]. Yields could consistently exceed 90% for various epoxide substrates. Noritaka Mizuno and colleagues proposed that monomeric tungstate could serve as an effective homogeneous catalyst, capable of activating CO_2_ to form adducts WO_4_-CO_2_ and WO_4_-(CO_2_)_n_ (n = 1, 2) [42]. The results indicated that CO_2_ could be immobilized with various structured aryl diamines, primary amines, propargyl alcohols, and propargyl amines, subsequently converting them into different fine chemical derivatives. These examples demonstrated the fundamental importance and great potential of the OM-ILs in CO_2_ catalysis.

Although great progress has been achieved, there are still several problems remaining in this area. Firstly, most of the OM-ILs catalysts are limited to the series of tungsten, with additional elements (such as Si, Mn, P) [43,44,45] incorporated to achieve catalytic functionality. Reports on non-tungsten OM-ILs for CO_2_ catalysis are quite rare. Secondly, most of the reports that utilize OM-ILs for CO_2_ activation are focused on the typical example of epoxide cyclization [46]. Consequently, it is quite urgent to design diversified OM-ILs for more CO_2_ conversion processes. Recently, Nicola Bragato and colleagues reported a molybdenum-based IL as a halogen-free catalyst for the catalytic conversion of CO_2_ [47]. Under conditions of 120 °C and 3 MPa, this catalyst could facilitate the cycloaddition reaction of various epoxides with CO_2_ to produce cyclic carbonates. However, the high temperature and high-pressure conditions required for this process presented unavoidable challenges. Additionally, this catalyst was still limited to using epoxides as substrates.

In this study, we shifted our focus to a different CO_2_ catalysis process. This process used propargyl alcohols as substrates to synthesize α-hydroxy ketones via a CO_2_-promoted hydration reaction. Molybdenum and tungsten belong to the same group in the periodic table of elements; hence the catalytic characteristics of molybdenum compounds may be similar to those of tungsten compounds, exhibiting excellent performance in CO_2_ capture and utilization. Compared to tungsten, molybdenum is more abundant in the earth and more cost-effective, which holds greater potential for practical application. Therefore, molybdate was selected as the core of the IL catalyst. Consequently, a dual-functional molybdate IL was designed and employed as the only catalyst for this process, without the usage of traditional Ag or Cu cations to activate the triple bonds in propargyl alcohols. To our knowledge, this is the first instance of an OM-IL catalyst being utilized for this reaction. During the experiments, the formation of MoO_4_-CO_2_ adducts was detected, which enhanced the catalytic efficiency of the reaction system. This system can achieve good yields of the target product under atmospheric CO_2_ conditions. Moreover, the [MoO_4_]^2−^ anions in these ILs originated from the wastewater from industrial processes that produce (NH_4_)_2_MoO_4_, implying a novel pathway to recycle and reuse this industrial wastewater.

## 2. Results

This section investigated the catalytic performance of various OM-ILs in the CO_2_-promoted hydration using 2-methyl-3-butyn-2-ol as the initial substrate (Table 1). The blank experiment revealed that the reaction could not proceed without catalysts (Table 1, entry 1). Additionally, no corresponding product was detected in the system when H_2_MoO_4_ and [N_4444_][Cl] were present individually or simultaneously (Table 1, entries 2–4). However, the addition of tetrabutylammonium molybdate ([N_4444_]_2_[MoO_4_]) to the system resulted in the generation of the target product (Table 1, entry 5). To demonstrate the superior catalytic performance of [N_4444_]_2_[MoO_4_] IL, further investigations were conducted on different anions and cations. Firstly, various valence states of molybdate ions were employed. However, none of them showed reactivity except [MoO_4_]^2−^ (Table 1, entries 5–7). In addition, [WO_4_]^2−^ gave a slightly lower yield of 55% (Table 1, entry 8). Consequently, the most effective anion was confirmed as [MoO_4_]^2−^. Subsequently, experiments testing different cations were performed. The ILs of [P_4444_]_2_[MoO_4_], [C_2_C_1_im]_2_[MoO_4_] and [DBUH]_2_[MoO_4_] showed considerable activity to the target reaction (Table 1, entries 9–11). On the contrary, the molybdate salts of Na_2_MoO_4_, K_2_MoO_4_, [NH_4_]_2_[MoO_4_] could not catalyze the reaction, even with the dissolution of these salts in the system by adding organic solvents such as DMF or CH_3_CN (Table 1, entries 12–14). Meanwhile, H_2_MoO_4_, Na_2_MoO_4_, K_2_MoO_4_, respectively, were added with [N_4444_][Lev], but no products formed (Table 1, entries 15–17). These results revealed the superiority of OM-ILs. Furthermore, the effects of different alkylammonium cation on catalytic activity were investigated (Table 1, entries 18–20). The results indicated that the catalytic activity increased with the growth of the carbon chain. This phenomenon may be attributed to the fact that the longer carbon chain weakens the electrostatic binding between the anion and cation, thereby enhancing the nucleophilicity of the anion [48]. In conclusion, [N_4444_]_2_[MoO_4_] was identified as the most effective catalyst in this investigation.

Upon validating [N_4444_]_2_[MoO_4_] as the most effective catalyst, we proceeded to investigate the reaction conditions, including reaction temperature, catalyst loading, and reaction time (Table 2). As the temperature increased from 40 °C to 100 °C, the yields significantly rose from 21% to 91% (Table 2, entries 1–4). Notably, a significant surge was observed around the temperature range of 60 °C to 80 °C, which could be attributed to the initiation of the dissolving process of the IL within this thermal range. After determining the optimal temperature as 100 °C, the catalyst loading was varied. With an increase in catalyst amount, the yields correspondingly increased and finally reached 91%. A comparison between entry 4 and entry 5 revealed that increasing the catalyst by an additional 25% resulted in only a 4% enhancement in yield. Consequently, the optimal catalyst loading was ascertained to be 1 equivalent. (Table 2, entries 4–8). Furthermore, it was observed that reducing the reaction time led to significant decreases in yields (Table 2, entries 9–11). Since the reaction yield had reached 87% at 1 bar of CO_2_, further experiments at higher pressures were not performed. In conclusion, the optimal reaction conditions were delineated as [N_4444_]_2_[MoO_4_] (1 equiv.), 100 °C, CO_2_ (0.1 MPa), and a reaction time of 12 h.

Following the optimization of the reaction conditions, we explored the reactivity of substrates featuring a variety of substituents (Table 3). The results indicated that the [N_4444_]_2_[MoO_4_] catalyst effectively facilitated the transformation of propargyl alcohols with diverse substituents into the targeted products. Notably, tertiary alcohols with alkyl (**1a**–**1f**), cycloalkyl (**1g**), and phenyl (**1h**) groups, which exhibited different steric hindered effects, achieved satisfactory yields. However, the secondary alcohol **1i** did not show activity for this reaction. This result is in concordance with the prior literature [49], which demonstrates that the Thorpe-Ingold effect plays a crucial role in the cyclization of CO_2_ with propargyl alcohols. In the case of tertiary alcohols, the dual alkyl substituents may compress the angle between the hydroxyl group and the triple bond, thereby facilitating cyclization with CO_2_. In contrast, secondary alcohols, lacking such alkyl substituents, do not exhibit this effect.

Furthermore, we conducted recycling experiments using **1a** as the model substrate. The results indicated yields of 87%, 69%, and 57% in recycle rounds 1–3. The gradually decreased yields might be attributed to the evaporation of substrates and the generation of inactive [Mo_2_O_7_]^2−^ during the reaction process [50].

**Figure 1:**
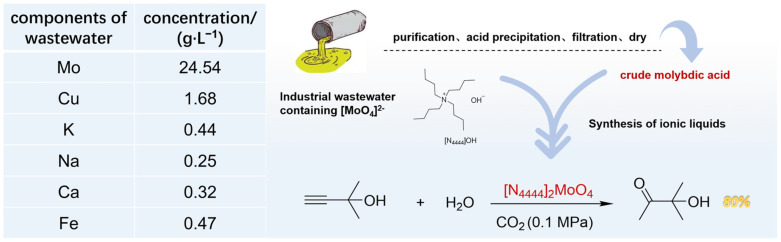
Reuse of ammonium molybdate wastewater.

For the reactions of CO_2_-promoted hydration of propargyl alcohols, most catalysts are typically purchased in high purity or synthesized using pure materials. This might be beneficial for mitigating the interference from impurities and for acquiring more precise catalytic data in the laboratory. Nevertheless, this has also limited further studies and applications of these catalysts in industries. In our previous report [51], we successfully used pigment waste as a source of metal catalysts combined with IL for efficient propargyl alcohols hydration catalysis, demonstrating a potential method for creating a catalytic system from recyclable waste. However, the IL component, as well as its synthetic materials in the system still required high purity. Therefore, in this work, we turned our attention to exploring a more general and cheap way for obtaining ILs. (NH_4_)_2_MoO_4_ is a pivotal catalyst for desulfurization processes in the petroleum refining industry. It is also a veterinary medicine in agricultural applications and a material in pigment production [52]. However, the industrial production of (NH_4_)_2_MoO_4_ is typically accompanied by the emission of wastewater containing Mo, Fe, Cu, etc. (as shown in Figure 1), posing significant environmental risks and leading to the wastage of molybdenum resources. Hence, we utilized this wastewater as a starting material for the synthesis of the [N_4444_]_2_[MoO_4_] IL. Initially, the wastewater underwent a series of steps, including purification, acid precipitation, filtration, and drying, to yield crude molybdic acid (Part 1 in Supplementary Materials). Subsequently, this crude molybdic acid was employed in the synthesis of [N_4444_]_2_[MoO_4_], following standard procedures (Figure 1). After obtaining the [N_4444_]_2_[MoO_4_] IL, we confirmed that its purity was around 92% through an ICP test, and the percentage of major metals in this catalyst was: Mo (13.57%), Cu (0.02%), Fe (0.004%). Promisingly, when this synthesized [N_4444_]_2_[MoO_4_] was applied to the target reaction, the catalytic yield reached 80%, suggesting that the [N_4444_]_2_[MoO_4_] catalyst showed remarkable tolerance to the catalytic environment. This experiment not only presented an innovative approach for the treatment and recycling of ammonium molybdate wastewater but also highlighted a feasible strategy for the large-scale implementation of CO_2_-promoted hydration processes.

## 3. Discussion

**Figure 2:**
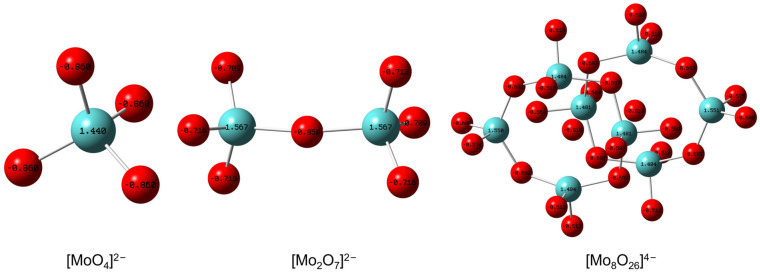
The calculation of molecular structures and NBO charges of oxygen atoms in various molybdates. The structures were calculated with DFT using the Gaussian 16 program package. Theory: B3LYP; basis sets: 6–31++G* (H, C, N, and O atoms) and LanL2DZ (Mo atoms). The red balls represented O atoms, and blue balls represented Mo atoms.

In the abovementioned study, the data in Table 1 highlighted the pivotal role of [MoO_4_]^2−^ in the reaction system. To understand why this anion performs better than other molybdate anions, we conducted an in-depth analysis. Initially, we optimized the structures of various molybdate anions using density functional theory (DFT). Subsequently, we compared the basicity of oxygen atoms within these polyoxometalates by examining the natural bond orbital (NBO) charges, as depicted in Figure 2. The oxygen atom in [MoO_4_]^2−^ exhibited a charge of −0.860, which was more negative than the charges in [Mo_2_O_7_]^2−^ and [Mo_8_O_26_]^4−^ (ranging from −0.858 to −0.503). This suggested that [MoO_4_]^2−^ possessed the strongest basicity among the molybdate anions studied. Based on previous studies, the basicity of IL anions is known to promote the activation of hydroxyl groups and CO_2_, which can significantly increase the reaction activity [53,54]. Consequently, this property of [MoO_4_]^2−^ could be the key factor contributing to its superior performance among the molybdate anions considered.

**Figure 3:**
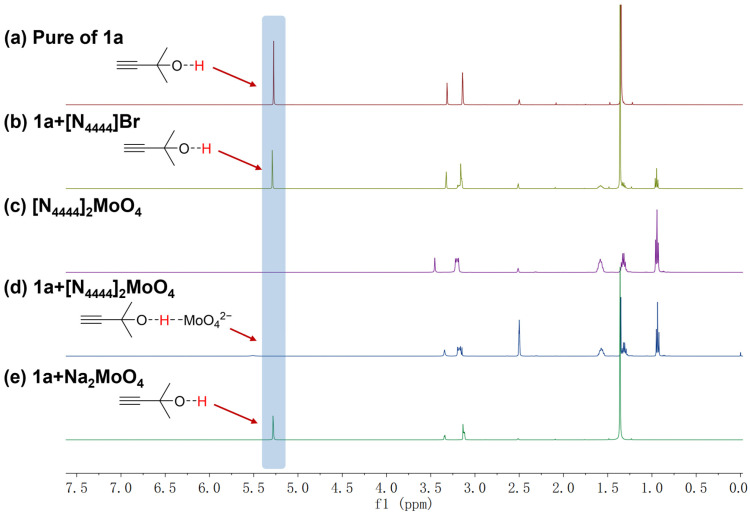
^1^H NMR of (**a**) pure **1a**; (**b**) **1a**/[N_4444_]Br (1:1); (**c**) pure [N_4444_]_2_MoO_4_; (**d**) **1a**/[N_4444_]_2_MoO_4_ (1:1); (**e**) **1a**/Na_2_MoO_4_ (1:1) in DMSO-*d*_6_. The chemical shifts of the hydroxyl groups in the five systems were indicated by shadow.

Subsequently, the function of [MoO_4_]^2−^ in the activation of hydroxyl groups within propargyl alcohols was investigated. According to the reported literature [17], this activation could be inferred from the shape and chemical shift in the hydroxyl signal peak in the ^1^H NMR spectrum of **1a**. As shown in Figure 3, five systems under identical conditions were prepared. For the pure substrate **1a**, the hydroxyl proton displayed a sharp peak at δ = 5.275 ppm, signifying its inactivated state (Figure 3a). The addition of [N_4444_]Br did not alter the shape or chemical shift in the hydroxyl peak in substrate **1a** (Figure 3b), indicating that [N_4444_]^+^ could not activate the hydroxyl group. However, upon the incorporation of [N_4444_]_2_MoO_4_, the characteristic peak of the hydroxyl group in substrate **1a** became broad and shifted. This observation conclusively demonstrated that [N_4444_]_2_MoO_4_ was capable of activating the hydroxyl group of **1a** (Figure 3c,d). On the other hand, the introduction of Na_2_MoO_4_ did not induce any alterations in the profile or position of the hydroxyl signal (Figure 3e), thereby suggesting that the [MoO_4_]^2−^ exhibited superior catalytic activity within ILs compared to its performance in traditional salts.

**Figure 4:**
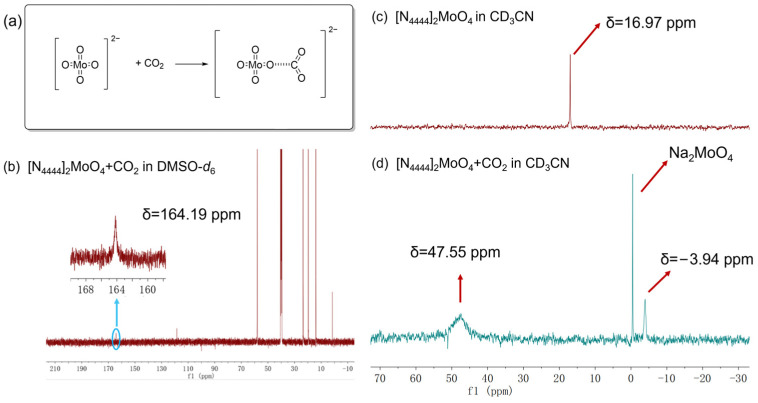
The possible mechanism of MoO_4_-CO_2_ adducts formation (**a**), ^13^C NMR spectrum of the MoO_4_-CO_2_ adduct (**b**), ^95^Mo NMR spectrum of [N_4444_]_2_MoO_4_ (**c**), and ^95^Mo NMR spectrum of the MoO_4_-CO_2_ adduct (**d**).

Subsequently, the activation function of [MoO_4_]^2−^ toward CO_2_ was examined. Based on the previous report [50], [MoO_4_]^2−^ might catch CO_2_ to form MoO_4_-CO_2_ adduct, suggesting a potentially activated state of CO_2_ (Figure 4a). To ascertain whether this [N_4444_]_2_[MoO_4_] IL could indeed react with CO_2_ to form this adduct, we conducted a series of experiments. Initially, [N_4444_]_2_[MoO_4_] was dissolved in anhydrous acetonitrile and transferred into a Schlenk flask, which was then sealed with a rubber stopper and exposed to CO_2_ for 12 h. Once the reaction finished, the solvent was carefully removed under low-temperature and vacuum conditions, yielding a white solid product. This white solid was subsequently analyzed using ^13^C NMR and ^95^Mo NMR spectroscopy. The ^13^C NMR spectrum revealed a distinct signal peak at δ = 164.19 ppm (Figure 4b), which aligns with the characteristic peak of the CO_2_ adduct reported in the literature [47,50], indicating the generation of MoO_4_-CO_2_ adduct. Additionally, distinct from the pure ^95^Mo NMR spectrum of [N_4444_]_2_[MoO_4_] (Figure 4c), a new signal peak emerged at δ = 47.55 ppm in the ^95^Mo NMR spectrum (Figure 4d), which was matched with the reported position of MoO_4_-CO_2_ [50]. These findings revealed that [N_4444_]_2_[MoO_4_] IL could react with CO_2_ to form a MoO_4_-CO_2_ adduct.

**Figure 5:**
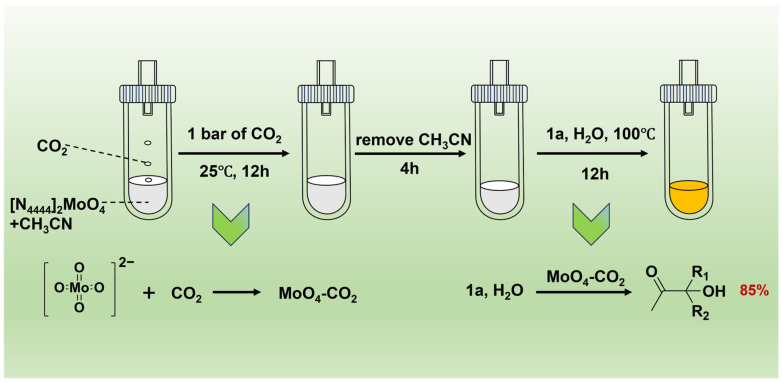
The experimental process of MoO_4_-CO_2_ adduct.

Conclusively, the catalytic activity of the MoO_4_-CO_2_ adduct in the hydration of propargyl alcohols was examined. The synthesis of the MoO_4_-CO_2_ adduct within the [N_4444_]_2_[MoO_4_] IL was carried out in accordance with the previously delineated methodology. Upon obtaining these adducts, propargyl alcohols and deionized water were introduced into the system, and the reaction was carried out following established protocols in Figure 5, in the absence of CO_2_. Upon completion, the resultant mixture was directly analyzed by ^1^H NMR. The result demonstrated that the reaction achieved a yield of 85%, which was close to the data obtained under standard conditions with CO_2_ gas as the carbon source. This finding confirmed that the MoO_4_-CO_2_ adduct independently catalyzed the hydration process with considerable activity, thereby demonstrating the superior catalytic capabilities of the [N_4444_]_2_[MoO_4_].

**Figure 6:**
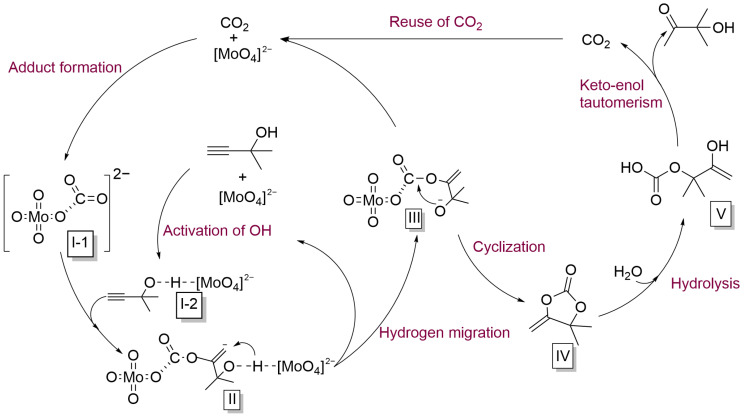
A possible catalytic mechanism of the CO_2_-promoted hydration of propargyl alcohols.

Based on previous reports and the experimental results obtained above [48], a possible catalytic mechanism for this system was proposed (Figure 6). Initially, under the influence of [N_4444_]_2_[MoO_4_], the hydroxyl group in the substrate was activated, enhancing the nucleophilicity of the oxygen atom in the hydroxyl group (**I-1**). Simultaneously, the interaction between the molybdate anion component and a molecule of CO_2_ led to the formation of adduct **I-2**, enabling the conversion of CO_2_ under mild conditions. Subsequently, the adduct **I-2** attacked the triple bond in **I-1**, forming intermediate **II**, which underwent a hydrogen migration from the hydroxyl group to generate intermediate **III**. Afterward, the negatively charged oxygen in intermediate **III** attacked the carbonyl carbon. The intramolecular cyclization occurred and carbonate **IV** was produced. Finally, the carbonate reacted with water to produce **V**, subsequently undergoing keto-enol tautomerism and releasing one molecule of CO_2_ to transform into the final target product.

## 4. Materials and Methods

### 4.1. Materials

Unless otherwise noted, all compounds involved in this study, including substrates and raw materials for the synthesis of ILs, were procured from Bide Pharm, Adamas, Aladdin, Macklin, and Alfa in China and were used without additional purification. The purity of the CO_2_ used for purging and reaction was 99.9%, supplied by Wuhan Xiangyun Industry and Trade Co., Ltd. (Wuhan, China). According to the literature, all ILs were synthesized by acid-base neutralization in deionized water or ethanol. The synthesized IL can be used directly without any further purification. Taking the synthesis of the [N_4444_]_2_[MoO_4_] IL as an example, molybdic acid (20 mmol) and deionized water (20 mL) were accurately weighed out, and they were placed into a 100 mL onion-shaped bottle. Under stirring conditions, tetrabutylammonium hydroxide (40 mmol) was added into the bottle. Then, the bottle was placed in a 25 °C water bath and the mixture was stirred for 6 h to ensure the complete dissolution of the molybdic acid. Afterwards, most of the solvent was removed by a vacuum rotary evaporator at 60 °C. Finally, the residual was continuously evaporated at 80 °C under a vacuum of oil for 8 h, thereby obtaining the [N_4444_]_2_[MoO_4_] IL [55,56].

### 4.2. Characterization Analysis

The ^1^H NMR spectra were recorded on a Bruker Avance III HD 500 MHz spectrometer, with either CDCl_3_ or DMSO-*d*_6_ solvents. Meanwhile, the ^13^C NMR spectra were recorded at 126 MHz in CDCl_3_ (δ = 77.23 ppm) or DMSO-*d*_6_ (δ = 39.50 ppm), with the solvent peaks serving as the internal references.

### 4.3. Experimental Procedures

Tetrabutylammonium Molybdate Catalyzed CO_2_-Promoted Hydration of Propargyl Alcohols.

Tetrabutylammonium molybdate ([N_4444_]_2_[MoO_4_] 2 mmol), 2-methyl-3-butyn-2-ol (2 mmol), and deionized water (4 mmol) were sequentially added to a 15 mL Schlenk flask. The gas atmosphere inside the Schlenk flask was rapidly exchanged three times with CO_2_. The mixture was then heated to react in a constant-temperature oil bath at 100 °C under 1 bar of CO_2_ and stirred for the required time. After the reaction finished, the mixture was extracted with ethyl ether (15 mL × 3). The upper layers were collected, and the solvent was removed under reduced pressure using a rotary evaporator to obtain the crude product. Further purification of the crude product was achieved by column chromatography (eluting with a petroleum ether/ethyl acetate solvent system at 100:1–20:1 *v*/*v*) to obtain the α-hydroxy ketones.

## 5. Conclusions

In summary, this study has designed a specific structured OM-IL ([N_4444_]_2_[MoO_4_]) that could independently catalyze the CO_2_-promoted hydration of propargyl alcohols to produce α-hydroxy ketones without additional additives. Compared to previously reported catalytic systems, this system is the first instance of an oxometallate-based IL catalyst being utilized for the CO_2_-promoted hydration of propargylic alcohols, converting a variety of propargyl alcohols into target products under atmospheric CO_2_ pressure. It is noteworthy that the [N_4444_]_2_[MoO_4_] could activate CO_2_ through the formation of MoO_4_-CO_2_ adducts. Furthermore, the molybdic acid used in the synthesis of the [N_4444_]_2_[MoO_4_] IL can originate from the wastewater of ammonium molybdate production, providing a potential method for the recycle and reuse of the wastewater. In the future, the potential applications of this catalytic system may extend to industrial processes, particularly in the conversion of CO_2_ into valuable chemicals. Additionally, it holds promise for environmental protection, especially in the area of repurposing molybdate wastewater. Future work will focus on optimizing the catalysts’ performance across diverse conditions, broadening their applicability to a wider scope of substrates, and evaluating their long-term stability and environmental sustainability.

## Figures and Tables

**Table 1:**
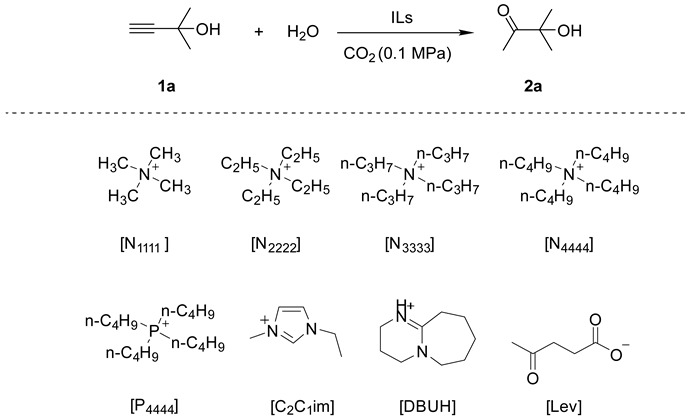
Screening of catalytic systems ^a^.

Entry	IL	Yield (%) ^b^
1	/	/
2	H_2_MoO_4_	/
3	[N_4444_][Cl]	/
4 ^c^	H_2_MoO_4_+[N_4444_][Cl]	/
5	[N_4444_]_2_[MoO_4_]	82
6	[N_4444_]_2_[Mo_2_O_7_]	/
7	[N_4444_]_4_[Mo_8_O_26_]	/
8	[N_4444_]_2_[WO_4_]	55
9	[P_4444_]_2_[MoO_4_]	49
10	[C_2_C_1_im]_2_[MoO_4_]	21
11	[DBUH]_2_[MoO_4_]	18
12 ^c^	Na_2_MoO_4_	/
13 ^c^	K_2_MoO_4_	/
14 ^c^	[NH_4_]_2_[MoO_4_]	/
15 ^c^	H_2_MoO_4_+[N_4444_][Lev]	/
16 ^c^	Na_2_MoO_4_+[N_4444_][Lev]	/
17 ^c^	K_2_MoO_4_+[N_4444_][Lev]	/
18	[N_1111_]_2_[MoO_4_]	20
19	[N_2222_]_2_[MoO_4_]	25
20	[N_3333_]_2_[MoO_4_]	34

^a^ Reaction conditions: **1a** (2 mmol), H_2_O (4 mmol), IL (2.5 mmol), 80 °C, CO_2_ (0.1 MPa), 12 h. ^b^ Yields were determined by ^1^H NMR spectroscopy using 1,3,5-trimethoxybenzene as the internal standard. ^c^ Add 1 mL of DMF or CH_3_CN as solvent.

**Table 2:**
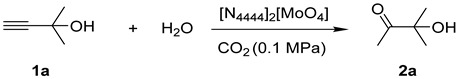
Effect of reaction conditions on the yields of **2a** ^a^.

Entry	IL (equiv.) ^c^	Temperature (°C)	Time (h)	Yield (%) ^b^
1	1.25	40	12	21
2	1.25	60	12	32
3	1.25	80	12	82
4	1.25	100	12	91
5	1	100	12	87
6	0.75	100	12	80
7	0.5	100	12	61
8	0.25	100	12	53
9	1	100	9	80
10	1	100	6	61
11	1	100	3	34

^a^ Reaction conditions: **1a** (2 mmol), H_2_O (4 mmol), CO_2_ (0.1 MPa). ^b^ Yields were determined by ^1^H NMR spectroscopy using 1,3,5-trimethoxybenzene as the internal standard. ^c^ Based on the amount of **1a**.

**Table 3:**
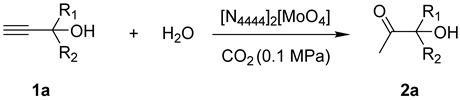
Substrates scope of the [N_4444_]_2_[MoO_4_] catalyst ^a^.

Substrate		Product		Time/h	Yield (%) ^b^
**1a**	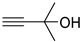	**2a**	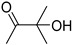	12	87 (69, 57) ^c^
**1b**	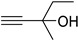	**2b**	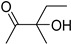	12	73
**1c**	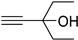	**2c**	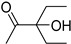	12	90
**1d**	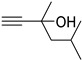	**2d**	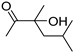	12	76
**1e**	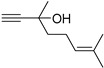	**2e**	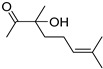	12	89
**1f**	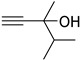	**2f**	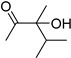	12	53
**1g**	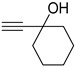	**2g**	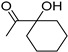	12	82
**1h**	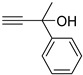	**2h**	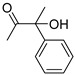	12	77
**1i**	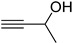	**2i**	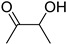	12	0

^a^ Reaction conditions: **1** (2 mmol), H_2_O (4 mmol), [N_4444_]_2_[MoO_4_] (2 mmol), CO_2_ (0.1 MPa), 100 °C, 12 h. ^b^ Yields were determined by ^1^H NMR spectroscopy using 1,3,5-trimethoxybenzene as the internal standard. ^c^ Yields in parentheses were obtained using recycled IL one and two times. IL was recycled by extracting the product with ether and drying under vacuum.

## Data Availability

Data are contained within the article and Supplementary Materials.

## Supplementary Materials

The supporting information can be downloaded at: https://www.mdpi.com/article/10.3390/ijms26010062/s1. References [57,58,59] are cited in the supplementary materials.
