# Supplementary material for: Oxometallate-Based Ionic Liquid Catalyzed CO2-Promoted Hydration of Propargylic Alcohols for α-Hydroxy Ketones Synthesis
Source: Int J Mol Sci. 2024 Dec 25;26(1):62. doi: 10.3390/ijms26010062 (PMC11719523; doi:10.3390/ijms26010062)
Supplement: Supplementary file 1 [file ijms-26-00062-s001.zip › ijms-3358801-supplementary.pdf]

Supplementary Files

For

Oxometallate-based Ionic Liquid Catalyzed CO_2-promoted Hydration of Propargylic Alcohols for α-Hydroxy Ketones Synthesis

Yuankun Wang, [a, b, #] Chongli Wang, [a, b, #] Weidong Lin, [a, b] Qin Wang, [a, b] Baisong Li, [a, b] Cheng Chen, [a] Ye Yuan, [*, a] Francis Verpoort [a]

[a] State Key Laboratory of Advanced Technology for Materials Synthesis and Processing, Wuhan University of Technology, Wuhan 430070, PR China.

[b] School of Materials Science and Engineering, Wuhan University of Technology, Wuhan 430070, PR China.

Corresponding author: Ye Yuan (Y. Y. : fyyuanye@whut.edu.cn)

[#] These two authors contribute equally to this work.

1. The treatment process of wastewater.

The ammonium molybdate wastewater mentioned in the article was prepared according to the ratio shown in **Fig. 1**, using raw material $(NH_4)_2MoO_4$ (2.5 g), $CuCl_2$ (0.176 g), KCl (0.042 g), NaCl (0.042 g), $CaCl_2$ (0.044 g), $FeCl_3$ (0.068 g), and deionized water (50 mL). The treatment process of ammonium molybdate wastewater includes four key steps: purification, acid precipitation, filtration, and drying. **Purification**: First, accurately measure 100 mL of the dark green transparent ammonium molybdate wastewater and pour it into a clean beaker. Adjust the pH to 6 by adding an appropriate amount of $NaHCO_3$. Then, place the beaker in a 50°C water bath and stir the solution continuously for 1 hour. After stirring, naturally filter the solution through a double-layer filter paper to obtain a clear purified liquid. **Acid precipitation**: Slowly add dilute hydrochloric acid to the purified liquid, reducing the pH to 1. The solvent was stirred at 70°C for 2 hours, then a white solid formed. **Filtration**: Cool the reaction mixture to room temperature and naturally filter it through a double-layer filter paper three times to ensure complete separation. **Drying**: Place the filtered solid in a vacuum drying oven and dry it for 4 hours at 80°C to obtain the desired crude molybdic acid product.

2. NMR spectra of the products and ionic liquid

All the products and ionic liquid mentioned below have already been characterized by ^{1}H NMR and ^{13}C NMR. The obtained data were matched with the previous reported publications of other researchers.

2a: ^{1}H NMR (500 MHz, DMSO-d_6) δ 5.22 (s, 1H), 2.16 (s, 3H), 1.18 (s, 6H) ppm. ^{13}C NMR (126 MHz, DMSO-d_6) δ 214.25, 76.20, 26.63, 24.66 ppm. These data are matched with the reported publication.[1]

^{1}H NMR of **2a** in DMSO-d_6

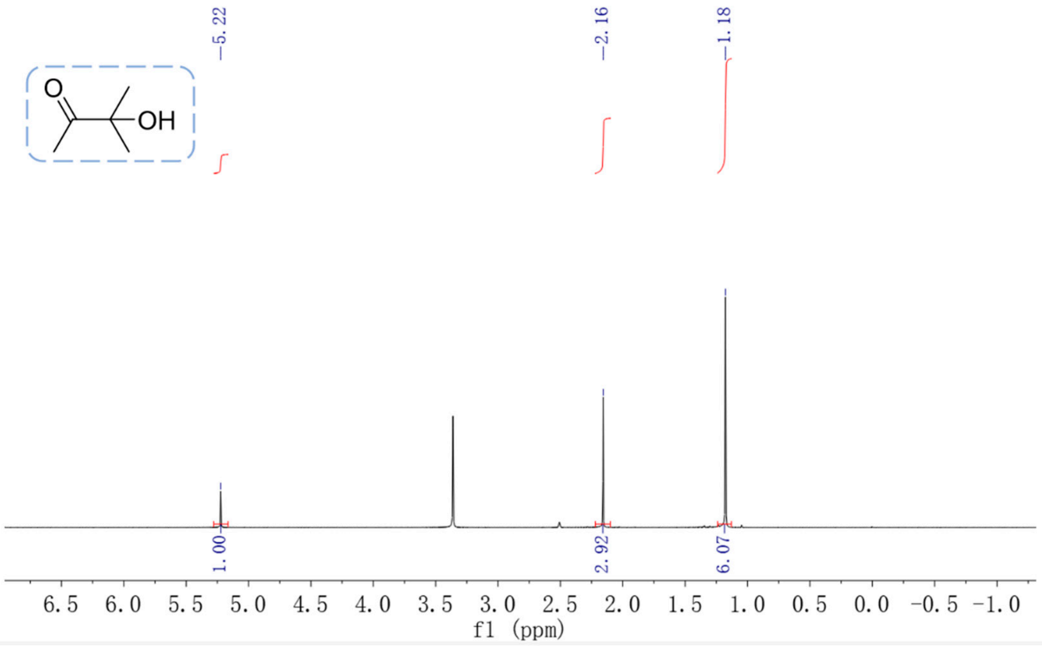

^{13}C NMR of **2a** in DMSO-d_6

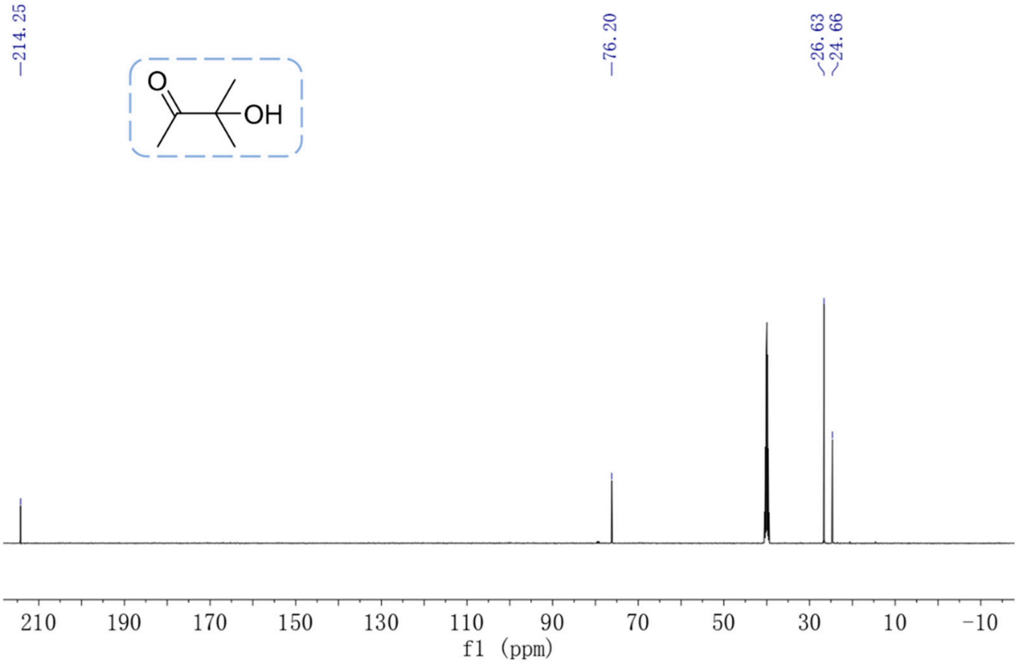

2b: ^{1}H NMR (500 MHz, DMSO-d_6) δ 5.04 (s, 1H), 2.14 (s, 3H), 1.64-1.44 (m, 2H), 1.13 (s, 3H), 0.76 (t, J = 7.5 Hz, 3H) ppm. ^{13}C NMR (126 MHz, DMSO-d_6) δ 214.52, 78.92, 32.16, 25.49, 24.53, 8.28 ppm. These data are matched with the reported publication.[1]

^{1}H NMR of **2b** in DMSO-d_6

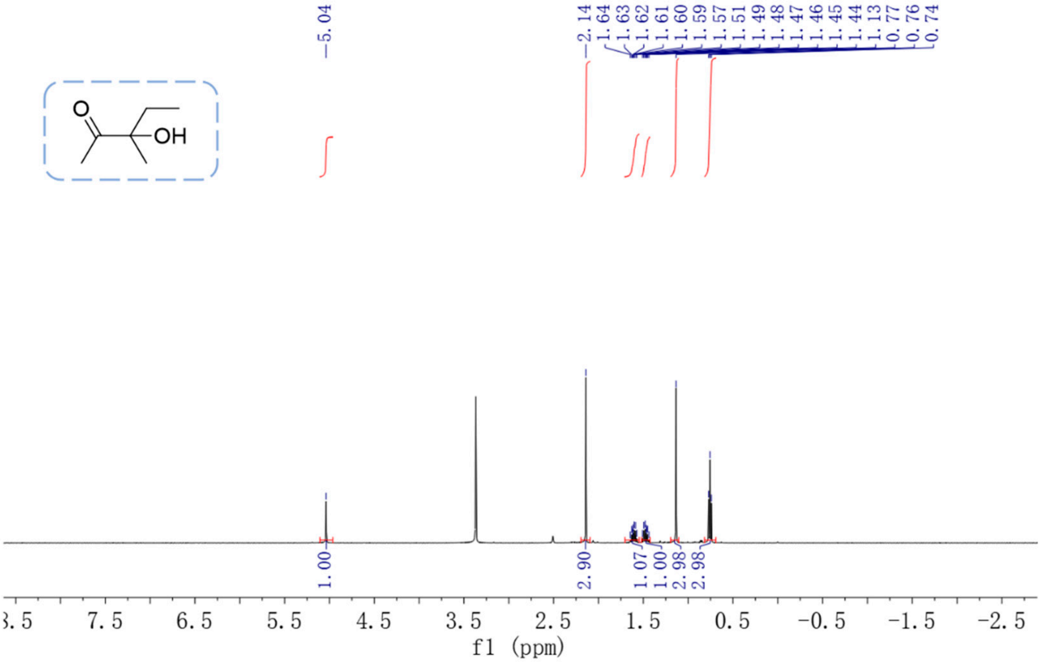

^{13}C NMR of **2b** in DMSO-d_6

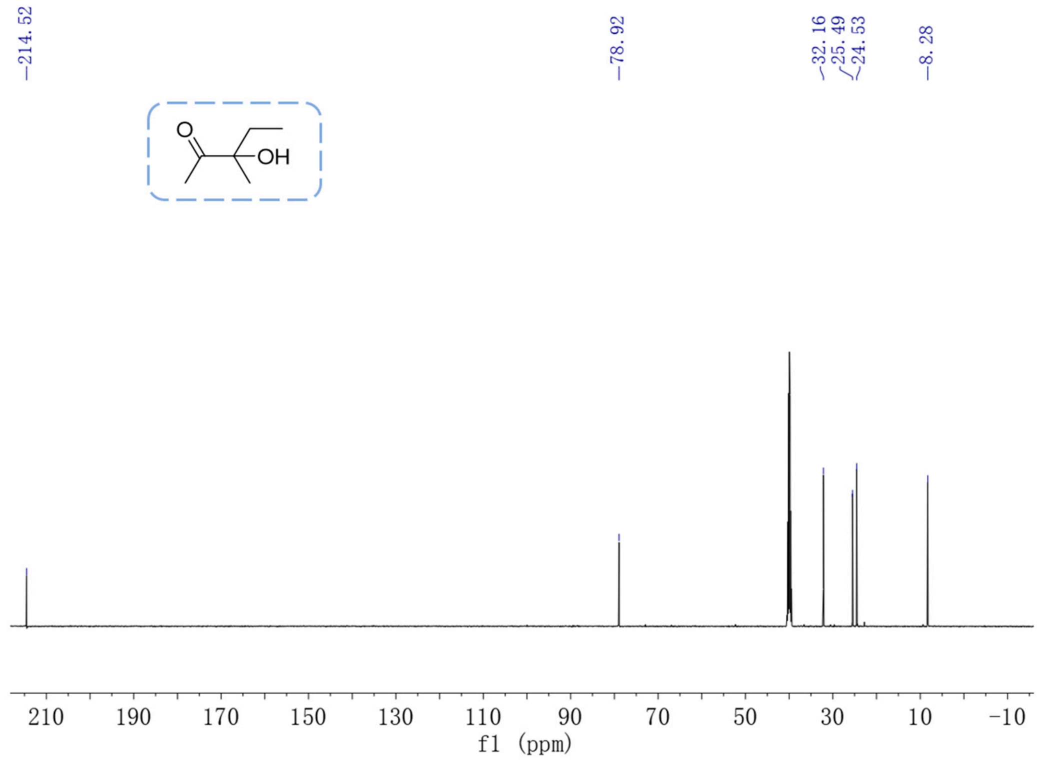

2c: ^{1}H NMR (500 MHz, DMSO-d_6) δ 4.77 (s, 1H), 2.12 (s, 3H), 1.65-1.46 (m, 4H), 0.73 (t, J = 7.5 Hz, 6H) ppm. ^{13}C NMR (126 MHz, DMSO-d_6) δ 214.48, 81.94, 30.87, 26.45, 8.13 ppm. These data are matched with the reported publication.[1]

^{1}H NMR of **2c** in DMSO-d_6

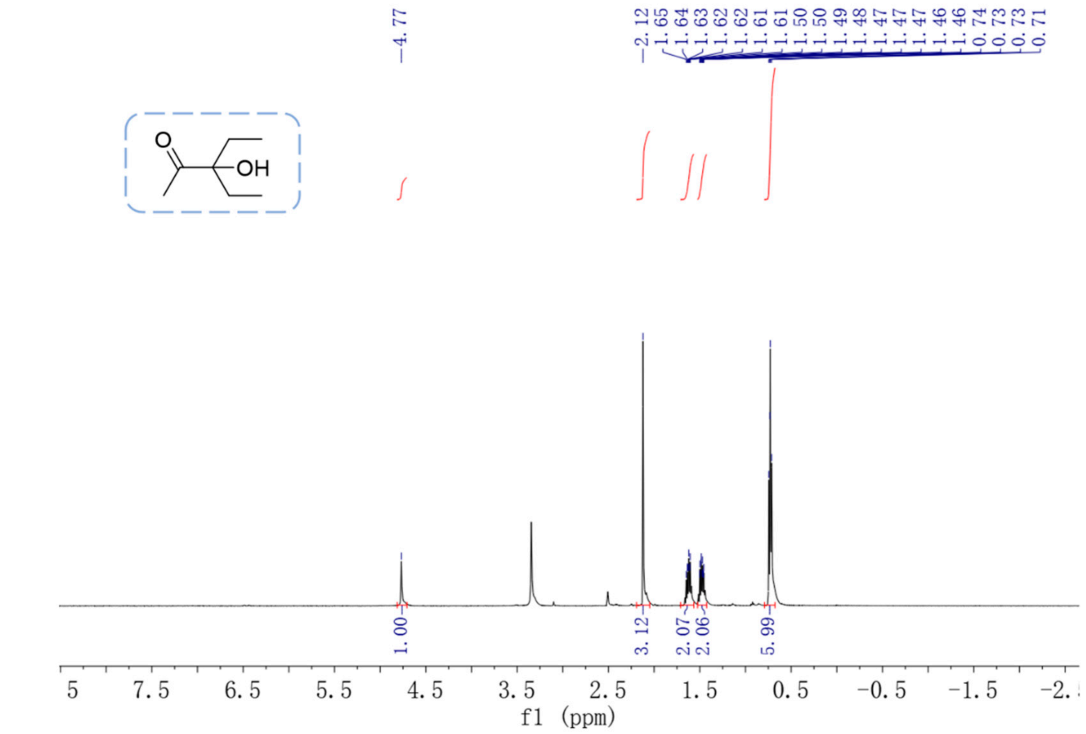

^{13}C NMR of **2c** in DMSO-d_6

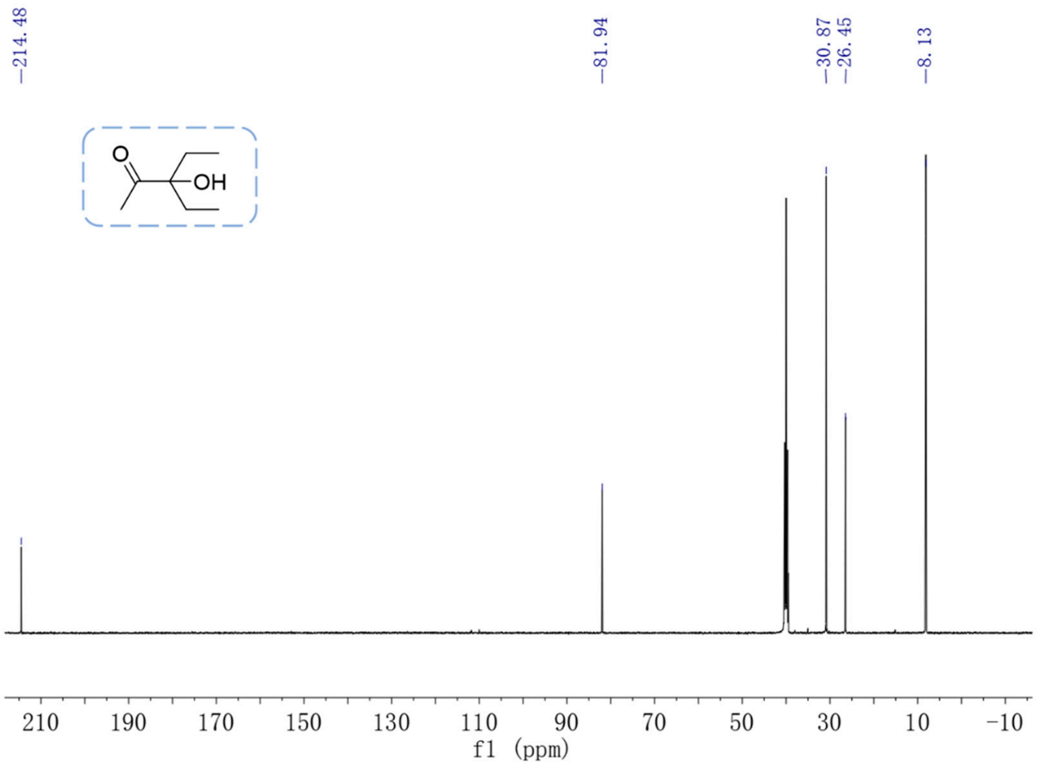

2d: ^{1}H NMR (500 MHz, DMSO-d_6) δ 5.00 (s, 1H), 2.17 (s, 3H), 1.69-1.56 (m, 2H), 1.42 (dd, J = 13.9, 5.6 Hz, 1H), 1.15 (s, 3H), 0.87 (d, J = 6.6 Hz, 3H), 0.79 (d, J = 6.6 Hz, 3H) ppm. ^{13}C NMR

(126 MHz, DMSO) δ 214.30, 78.49, 47.57, 25.94, 25.08, 24.24, 23.71 ppm. These data are matched with the reported publication.[1]

^{1}H NMR of **2d** in DMSO-d_6

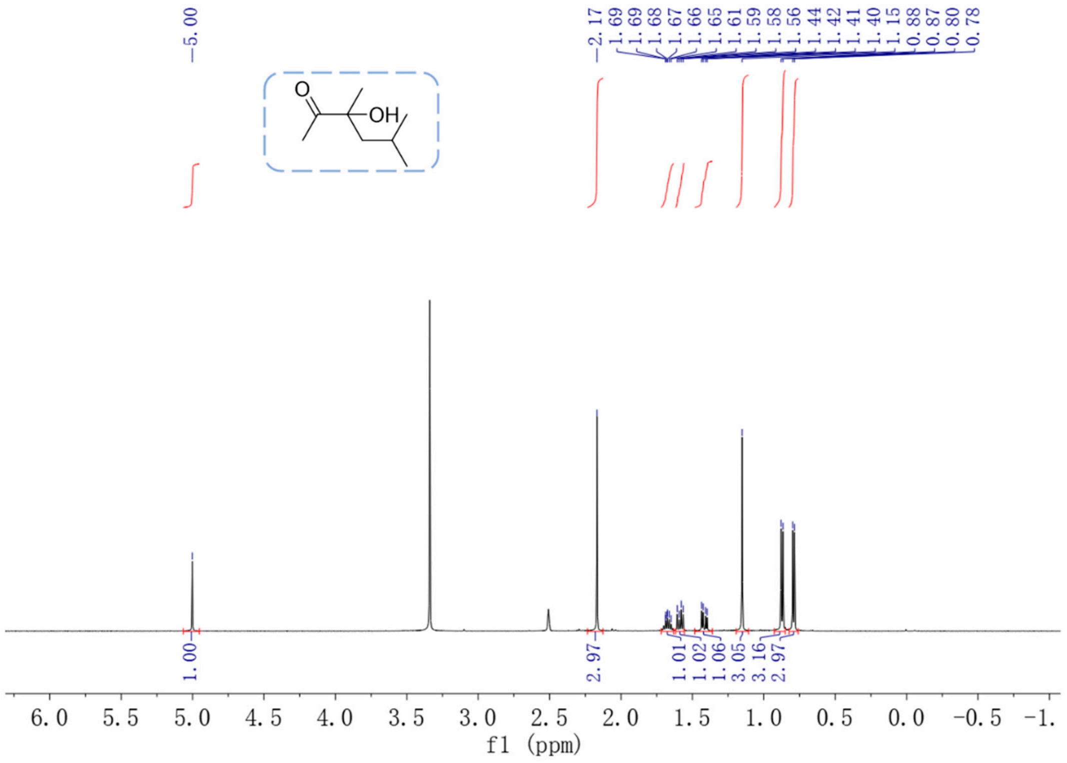

^{13}C NMR of **2d** in DMSO-d_6

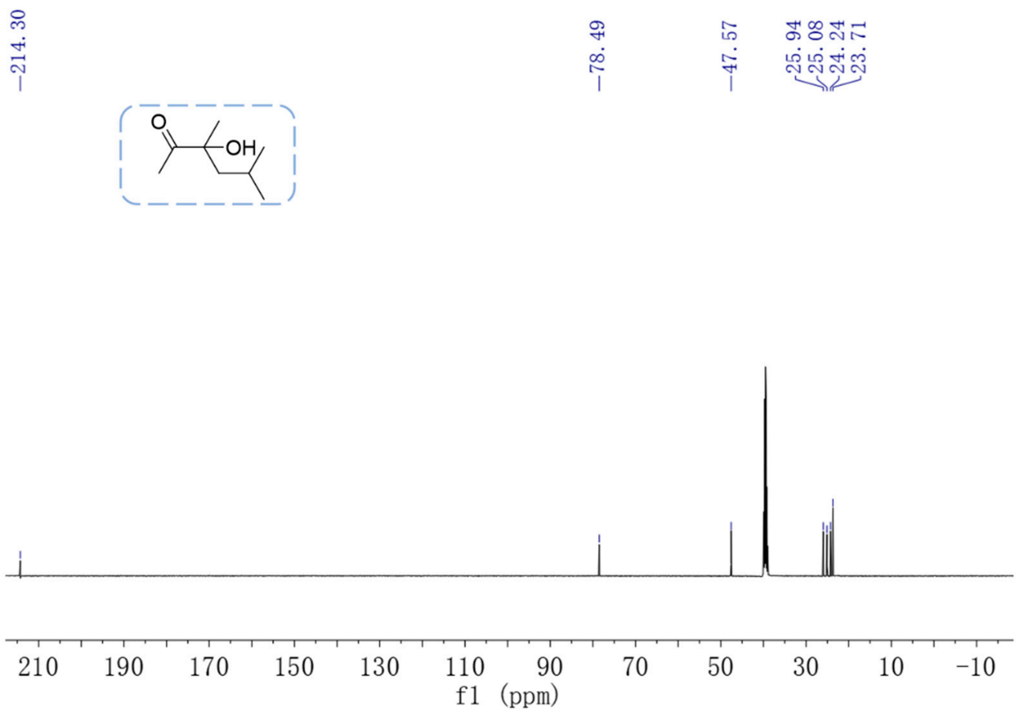

2e: ^{1}H NMR (500 MHz, $CDCl_3$) δ 5.06 (t, *J* = 7.6 Hz, 1H), 3.87 (s, 1H), 2.23 (s, 3H), 2.13-2.06 (m 1H), 1.84-1.74 (m, 3H), 1.68 (s,3H), 1.59 (s, 3H), 1.37 (s, 3H) ppm. ^{13}C NMR (126 MHz, $CDCl_3$) δ 212.03, 132.42, 123.37, 78.61, 39.41, 25.50, 23.53, 22.13, 17.51 ppm. These data are matched with the reported publication.[2]

^{1}H NMR of **2e** in $CDCl_3$

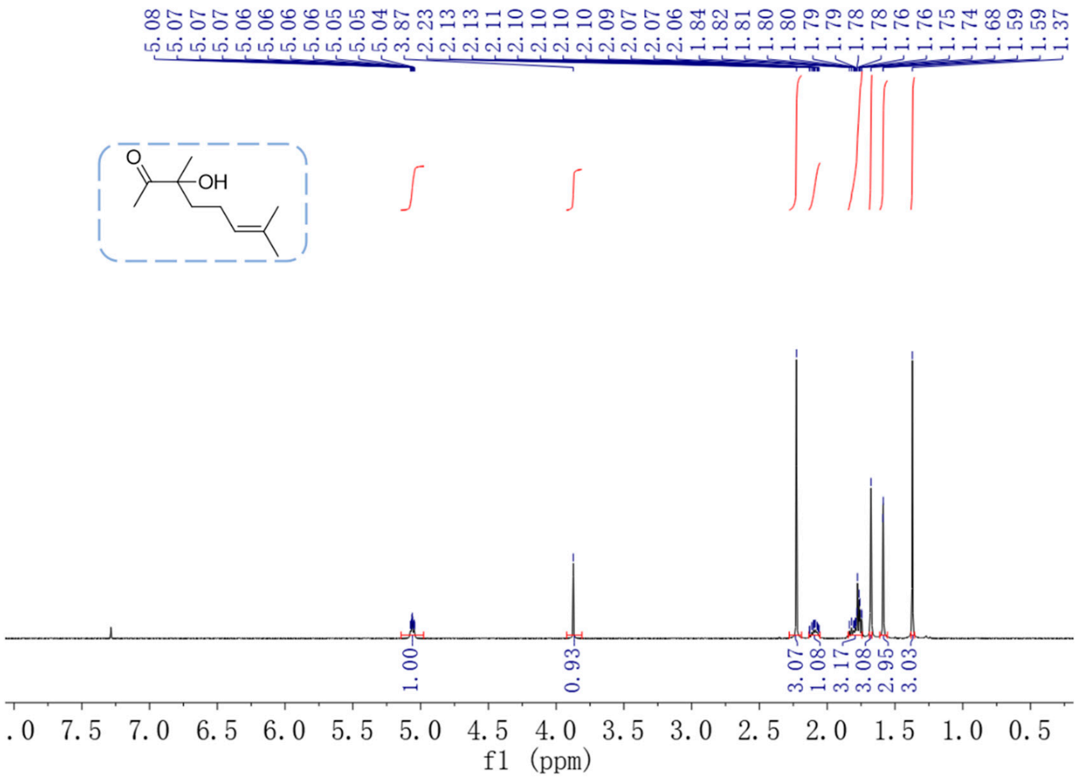

^{13}C NMR of **2e** in $CDCl_3$

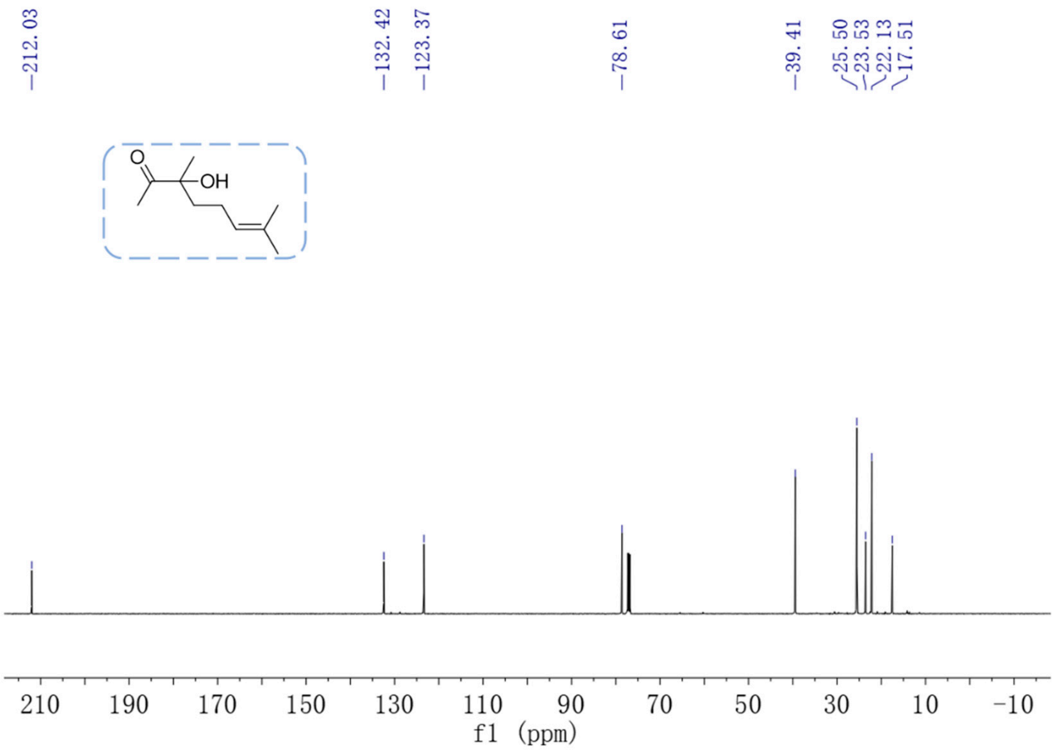

2f: ^{1}H NMR (500 MHz, DMSO-d_6) δ 4.87 (s, 1H), 2.12 (s, 3H), 1.87 (dt, J = 13.6, 6.8 Hz, 1H), 1.07 (s, 3H), 0.77 (dd, J = 24.3, 6.8 Hz, 6H). ^{13}C NMR (126 MHz, DMSO) δ 214.84, 80.79, 34.53, 25.82, 2.05, 17.31, 16.77. These data are matched with the reported publication.[3]

^{1}H NMR of **2f** in DMSO-d_6

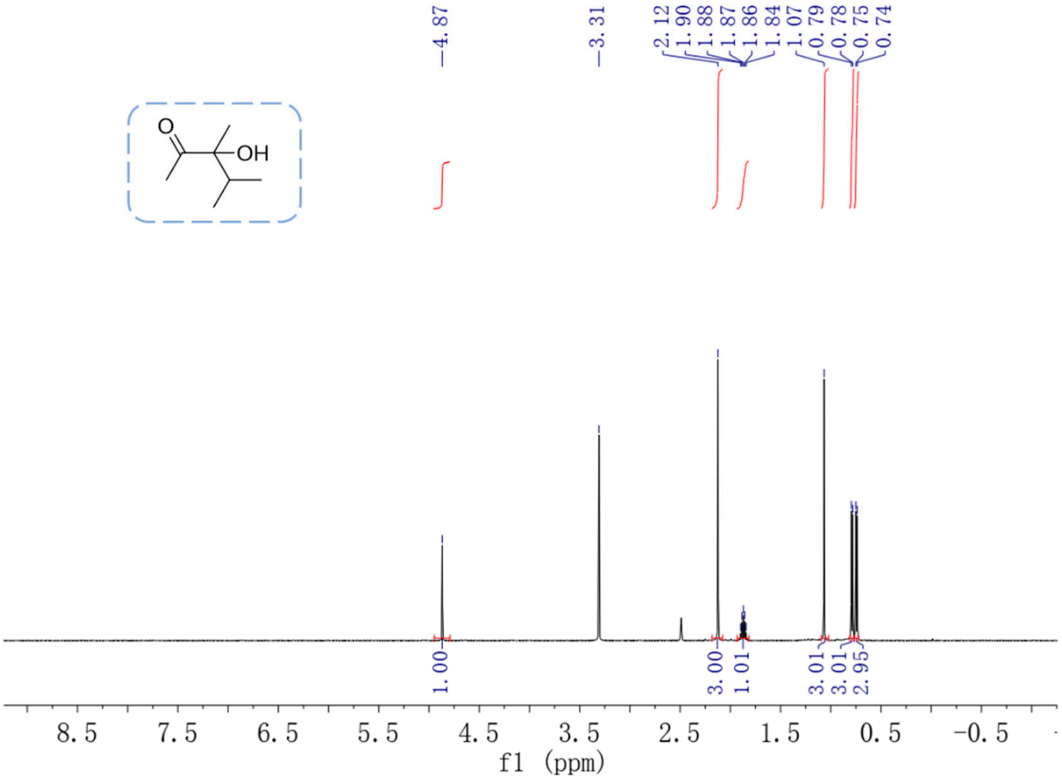

^{13}C NMR of **2f** in DMSO-d_6

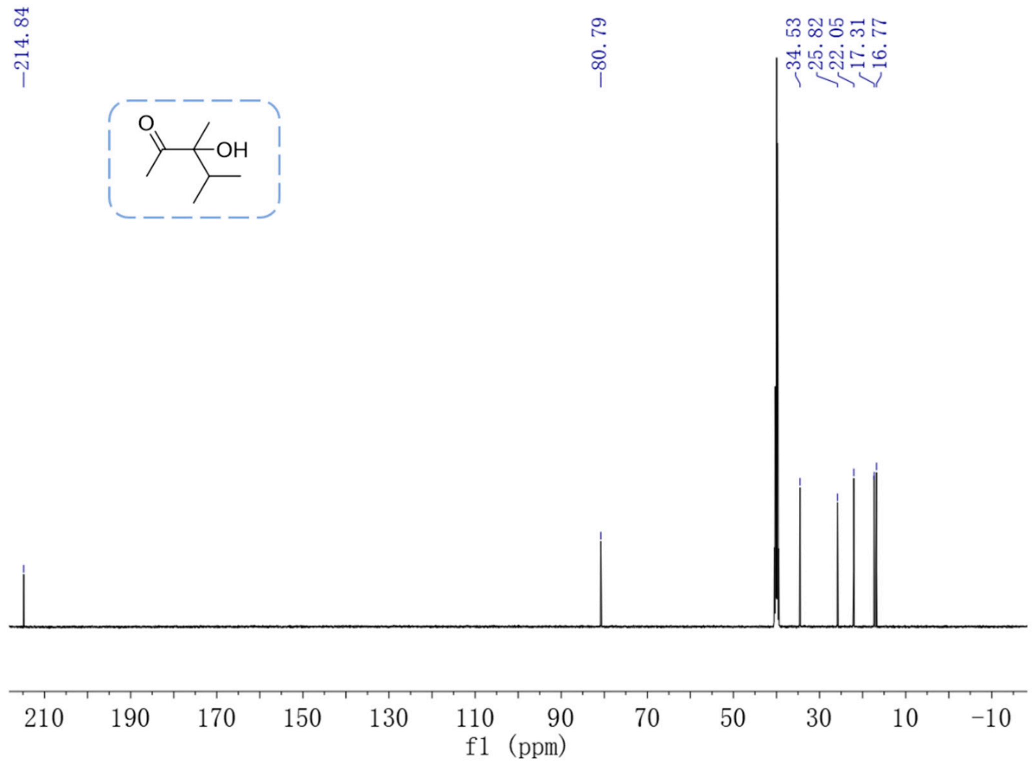

2g: ^{1}H NMR (500 MHz, DMSO-d_6) δ 5.08 (s, 1H), 2.17 (s, 3H), 1.61-1.16 (m, 10H) ppm. ^{13}C NMR (126 MHz, DMSO-d_6) δ 214.51, 77.27, 33.27, 25.46, 24.82, 21.22 ppm. These data are matched with the reported publication.[1]

^{1}H NMR of **2g** in DMSO-d_6

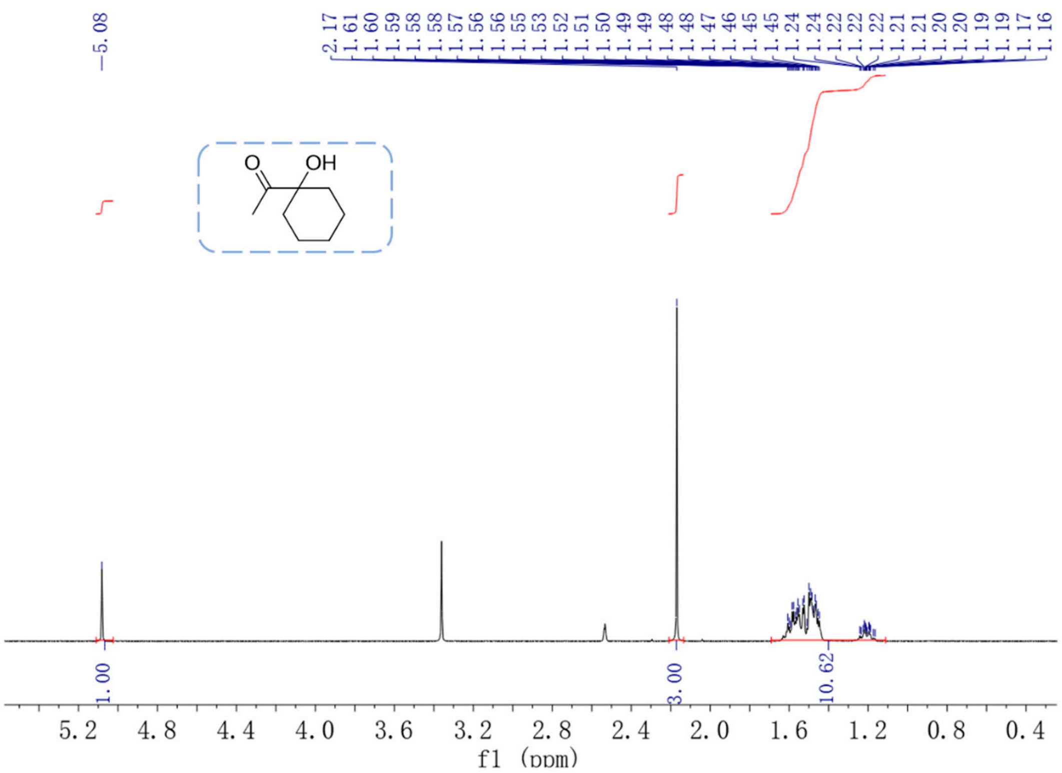

^{13}C NMR of **2g** in DMSO-d_6

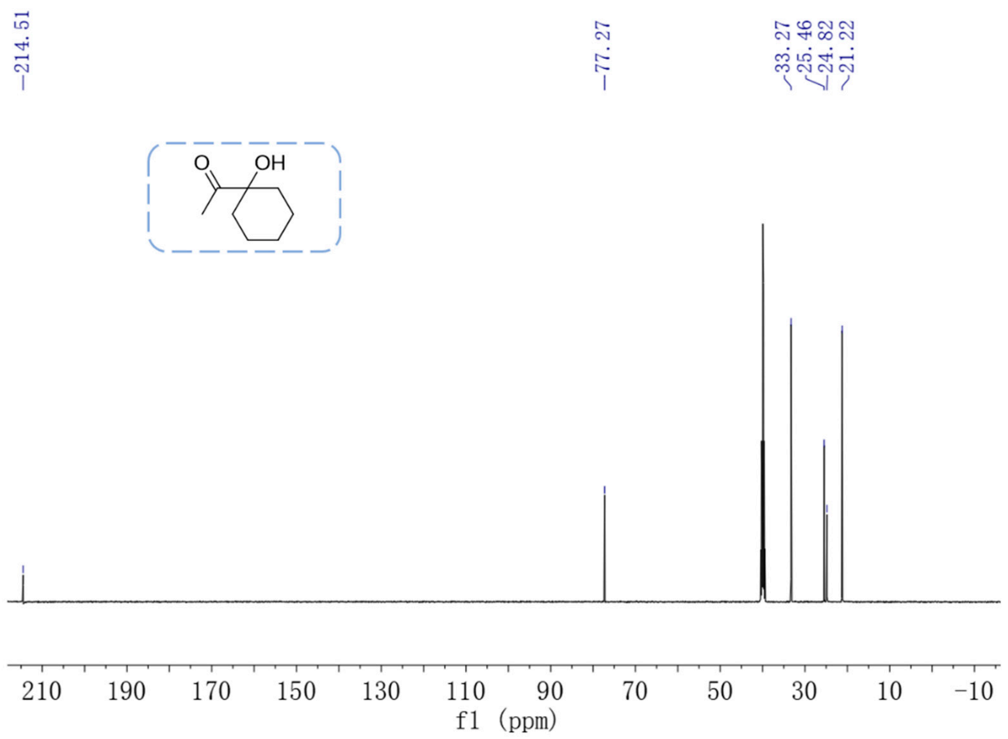

2h: ^{1}H NMR (500 MHz, DMSO-d_6) δ 7.37 (dd, J = 8.4, 1.3 Hz, 2H), 7.29 (t, J = 7.7 Hz, 2H), 7.27-7.20 (m, 1H), 5.97 (s, 1H), 1.96 (s, 3H), 1.45 (s, 3H) ppm. ^{13}C NMR (126 MHz, DMSO-d_6) δ 210.17,

143.21, 128.10, 127.05, 124.92, 79.62, 25.97, 24.15 ppm. These data are matched with the reported publication.[1]

^{1}H NMR of **2h** in DMSO-d_6

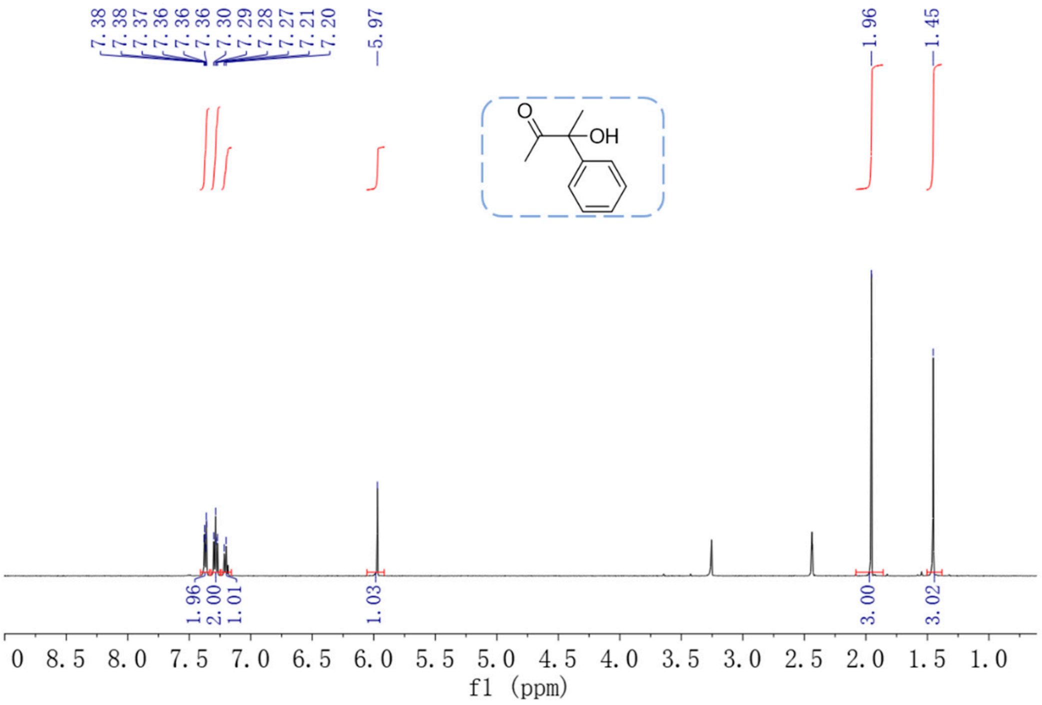

^{13}C NMR of **2h** in DMSO-d_6

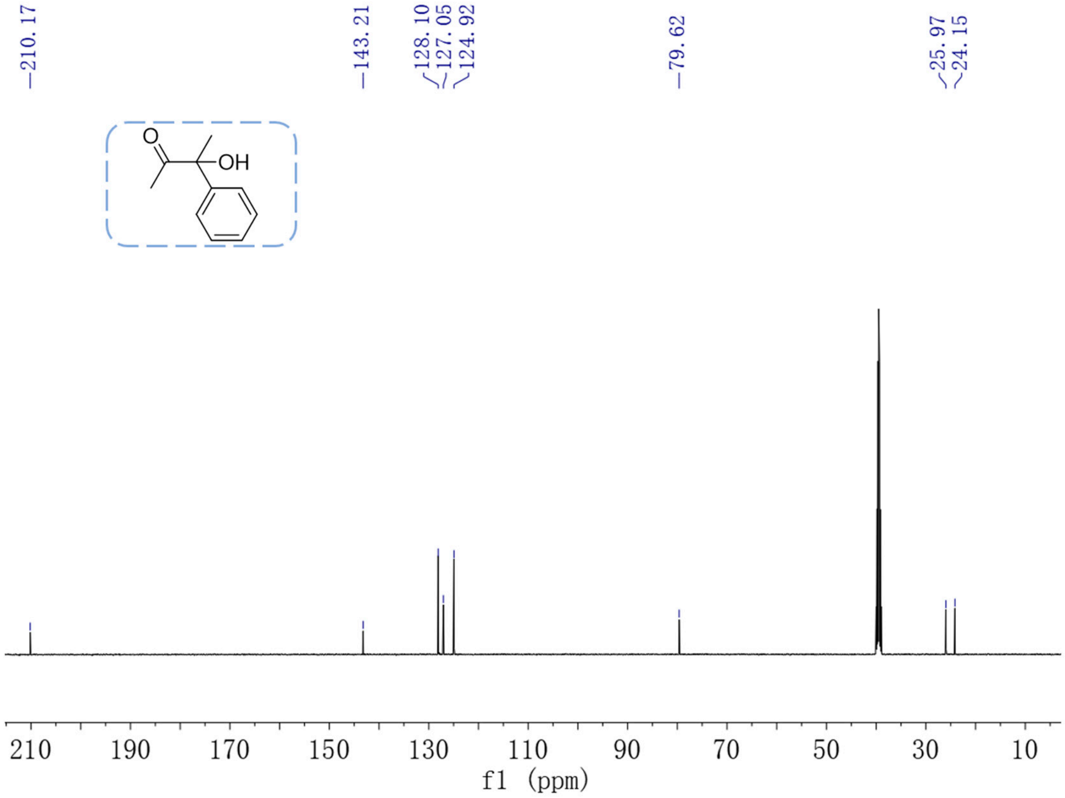

$[N_{4444}]_2MoO_4$: ^1H NMR (500 MHz, $CDCl_3$) δ (ppm): 3.44-3.40 (m, 8H), 1.73-1.66 (m, 8H), 1.51-1.44 (m, 16H), 1.00 (t, J = 7.4 Hz, 12H) ppm. ^{13}C NMR (126 MHz, $CDCl_3$) δ 58.83, 24.09, 19.78, 13.74 ppm. These data are matched with the reported publication.[4]

^1H NMR of $[N_{4444}]_2MoO_4$ in $CDCl_3$

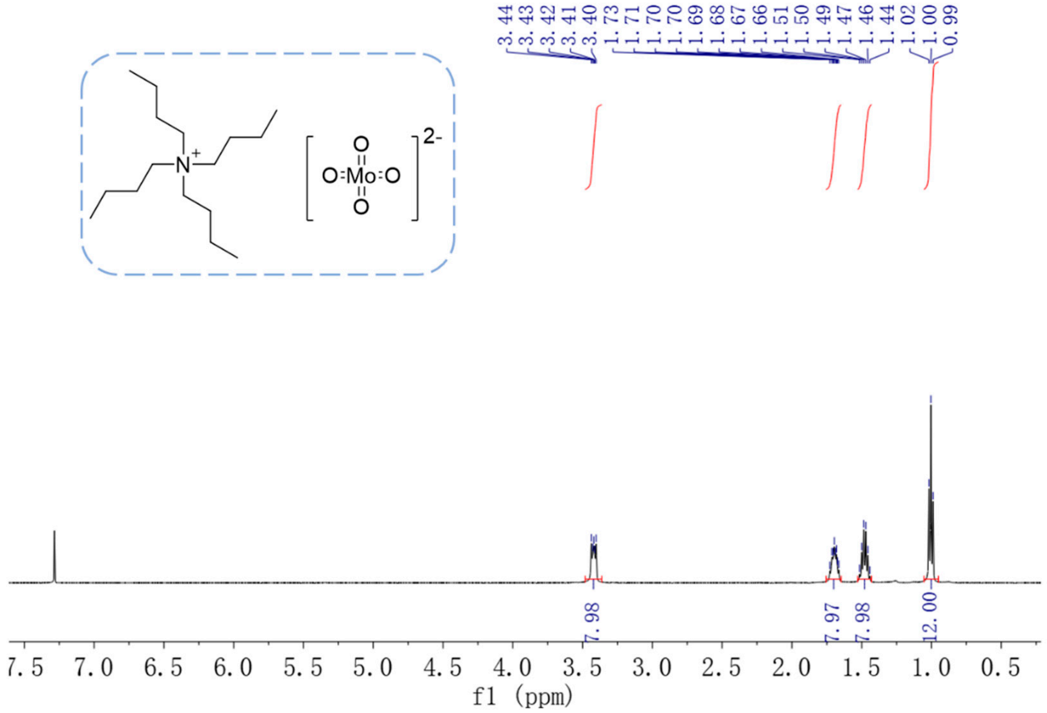

^{13}C NMR of $[N_{4444}]_2MoO_4$ in $CDCl_3$

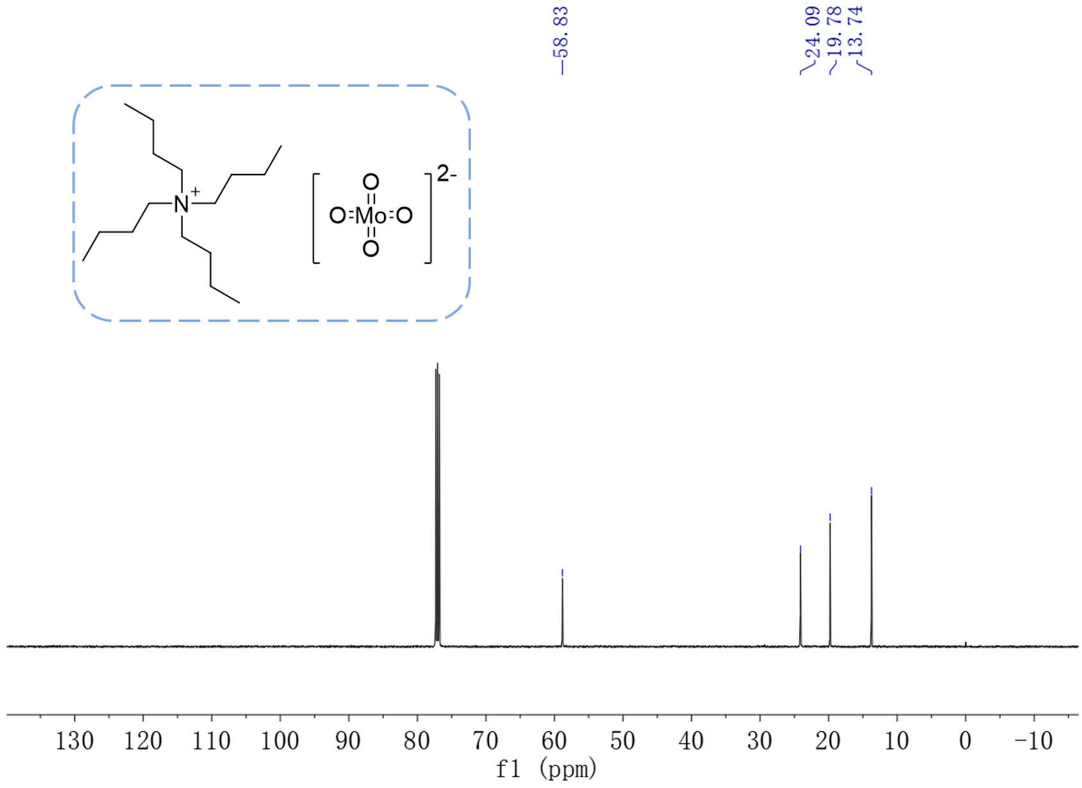

3. References

1. Zhao, Y.; Yang, Z.; Yu, B.; Zhang, H.; Xu, H.; Hao, L.; Han, B.; Liu, Z., Task-

specific ionic liquid and CO_2-cocatalysed efficient hydration of propargylic alcohols to α-hydroxy ketones. *Chem. Sci.* **2015,** *6* (4), 2297-2301.
2. Cardillo, G.; Orena, M.; Porzi, G.; Sandri, S.; Tomasini, C., Novel synthesis of .alpha.-hydroxy ketones and .gamma.- or .delta.-keto esters from cyclic iodo carbonates and iodo lactones. *J. Org. Chem.* **1984,** *49* (4), 701-703.
3. Davis, B. R.; Rewcastle, G. W.; Woodgate, P. D., Clemmensen reduction. Part 7. Acid-catalysed opening of cyclopropane-l,2-diols. *J. Chem. Soc., Perkin Trans. 1* **1979,** (0), 2820-2825.
4. Bragato, N.; Perosa, A.; Selva, M.; Fiorani, G.; Calmanti, R., Molybdate ionic liquids as halide-free catalysts for CO_2 fixation into epoxides. *Green Chem.* **2023,** *25* (12), 4849-4860.
